# Functional genomic analysis and neuroanatomical localization of miR-2954, a song-responsive sex-linked microRNA in the zebra finch

**DOI:** 10.3389/fnins.2014.00409

**Published:** 2014-12-16

**Authors:** Ya-Chi Lin, Christopher N. Balakrishnan, David F. Clayton

**Affiliations:** ^1^Genomics of Neural and Behavioral Plasticity Theme, Institute for Genomic Biology, University of IllinoisUrbana-Champaign, IL, USA; ^2^Department of Cell and Developmental Biology, University of IllinoisUrbana-Champaign, IL, USA; ^3^Department of Biology and Center for Biodiversity, East Carolina UniversityGreenville, NC, USA; ^4^Department of Biological and Experimental Psychology, School of Biological and Chemical Sciences, Queen Mary University of LondonLondon, UK

**Keywords:** microRNA, zebra finch, RNAseq analysis, *In situ* hybridization, fluorescence, MAPK and ERK signaling, NR4A3, cell line

## Abstract

Natural experience can cause complex changes in gene expression in brain centers for cognition and perception, but the mechanisms that link perceptual experience and neurogenomic regulation are not understood. MicroRNAs (miRNAs or miRs) have the potential to regulate large gene expression networks, and a previous study showed that a natural perceptual stimulus (hearing the sound of birdsong in zebra finches) triggers rapid changes in expression of several miRs in the auditory forebrain. Here we evaluate the functional potential of one of these, miR-2954, which has been found so far only in birds and is encoded on the Z sex chromosome. Using fluorescence *in situ* hybridization and immunohistochemistry, we show that miR-2954 is present in subsets of cells in the sexually dimorphic brain regions involved in song production and perception, with notable enrichment in cell nuclei. We then probe its regulatory function by inhibiting its expression in a zebra finch cell line (G266) and measuring effects on endogenous gene expression using Illumina RNA sequencing (RNA-seq). Approximately 1000 different mRNAs change in expression by 1.5-fold or more (adjusted *p* < 0.01), with increases in some but not all of the targets that had been predicted by Targetscan. The population of RNAs that increase after miR-2954 inhibition is notably enriched for ones involved in the MAP Kinase (MAPK) pathway, whereas the decreasing population is dominated by genes involved in ribosomes and mitochondrial function. Since song stimulation itself triggers a decrease in miR-2954 expression followed by a delayed decrease in genes encoding ribosomal and mitochondrial functions, we suggest that miR-2954 may mediate some of the neurogenomic effects of song habituation.

## Introduction

Zebra finches are songbirds that communicate using learned vocalizations (Immelmann, 1969; Miller, 1979; Clayton, 1988), and have become important model organisms for studying the neural and genomic mechanisms of social learning, memory, and sex-linked behavior (Replogle et al., 2008; Robinson et al., 2008; Clayton et al., 2009; Clayton, 2013). Both the act of singing, and the experience of hearing other birds sing, can elicit complex changes in gene expression in discrete regions of the higher forebrain (reviewed in Clayton, 2013). Hundreds of genes (at least) are involved in these responses, with some genes changing their expression within minutes after an experience, whereas other changes only follow after several hours or even days (Dong et al., 2009). These observations define a new frontier in understanding how experiences are encoded in the brain and raise new questions about how these complex shifts in gene expression are orchestrated (Clayton, 2013).

MicroRNAs (miRNAs or miRs) comprise a family of non-coding RNAs (ncRNAs) that may orchestrate the expression of multiple genes via direct interactions with mRNAs. The suite of miRs expressed in the zebra finch brain has recently been described (Li et al., 2007; Warren et al., 2010; Gunaratne et al., 2011; Luo et al., 2012; Shi et al., 2013). Some of these miRs are regulated themselves in response to the sound of song playbacks, suggesting they could have a functional role in the neurogenomic networks involved in song perception and songbird behavior (Gunaratne et al., 2011). Here we focus on one of these song-regulated miRs, mir-2954 (Gunaratne et al., 2011), which is of particular interest for several reasons. The gene for miR-2954 is on the avian Z sex chromosome (females: ZW, males ZZ) and has not been found outside the avian lineage. It produces at least three different products from both strands, with significantly higher expression in males (ZZ) compared to females (ZW) (Gunaratne et al., 2011). Moreover, evidence suggests that miR-2954 may respond to song differently in the two sexes, clearly down-regulated in females, but mildly up-regulated in males 30 min after hearing song (Gunaratne et al., 2011).

To evaluate the potential of miR-2954 for a neural regulatory function in songbirds, we set out here to answer two questions. First, where is miR-2954 expressed in the brain? Because it is sex-linked, we focused in particular on the sexually dimorphic nuclei of the telencephalic song control circuit, and considered both broad regional expression and cellular localization. Second, what are the consequences of suppressing miR-2954 expression—does this alter the expression of other genes? Although mRNA targets have been predicted for mir-2954 using computational approaches (e.g., TargetScan; Lewis et al., 2005; Gunaratne et al., 2011), there is as yet no direct evidence that a change in miR-2954 expression will cause a change in the expression of other genes. To test this, we took advantage of a zebra finch cell line (Itoh and Arnold, 2011) in which gene expression patterns have recently been analyzed using RNA sequencing (RNA-seq) (Balakrishnan et al., 2012). As our results identified a large number of genes that are sensitive to miR-2954 inhibition, we then applied statistical annotation tools to assess the potential functional significance and to compare the response to miR-2954 inhibition with the previously described neurogenomic response to song playbacks (Dong et al., 2009). Our study represents one of the first functional analyses of gene network manipulation in the songbird system, and qualifies the capacity of miR-2954 to influence the structure of gene expression networks involved in song perception.

## Materials and methods

### Animals

All procedures involving animals were conducted with protocols approved by the University of Illinois Institutional Animal Care and Use Committee. Zebra finches used in this study were adults (older than 90 days after hatching) and obtained from aviaries maintained at the University of Illinois. The birds were raised in a standard breeding aviary and learned their songs under normal social conditions (i.e., by their parents or other adult birds in the breeding colony).

To compare expression of miR-2954 in different tissues and between the sexes, six birds including three females and three males were sacrificed by rapid decapitation. A total of nine tissues, whole brain, muscle, heart, liver, lung, spleen, gonad, kidney, and adrenal gland, were dissected from each bird, frozen on dry ice and stored in a -80 freezer until RNA purification. To map miR-2954 in the brain using *in situ* hybridization, another seven male and six female birds were exposed to song playback or silence as in previous studies, using the same equipment (Dong et al., 2009; Gunaratne et al., 2011). Each bird was put individually into a sound isolation chamber for 18 h on the first day, and on the second day those in the song group heard 30 min of a song not heard previously (“novel song”). Matched controls collected in parallel heard no song playback (“silence”). Birds were sacrificed in song-silence pairs, so that 5 min before the end of the song playback to one bird, a bird in the silence group was sacrificed by rapid decapitation, and its whole brain was removed, placed in Tissue-Tek O.C.T. compound, and rapidly frozen on dry ice. Immediately after that, the same dissection procedure was performed to collect the brain from the matched song-stimulated bird. At the end of the song stimulation procedure, all brains were transferred and stored at −80°C until they were sectioned on a cryostat.

### Cell culture and miR-2954 inhibition

Many miRs function by binding to complementary sequences typically found in the 3′-UTRs of target mRNAs, triggering degradation of these mRNAs and thus suppressing the expression of the target genes at a post-transcriptional level. We previously noted the presence of predicted binding sites for miR-2954 in the 3′-UTR of the song-regulated NR4A3 mRNA (Warren et al., 2010), and we identified eight other song-regulated mRNAs that also carry predicted miR-2954 binding sites in their 3′-UTRs (Gunaratne et al., 2011). To test whether changes in miR-2954 expression affect the levels of these or other mRNAs, we used manufacturers' procedures to transfect (Oligofectamine, Invitrogen) a synthetic sequence-specific miR-2954 inhibitor (Meister et al., 2004; Schratt et al., 2006) into a male zebra finch cell line (G266), grown as previously described (Itoh and Arnold, 2011; Balakrishnan et al., 2012). The miR inhibitor, obtained from Dharmacon RNAi Technology (Lafayette, CO), is a single-stranded RNA molecule bearing a proprietary chemical modification and secondary structures in the flanking region to suppress the function of miR-miRISC complex through reverse complementarity to miR sequence (Vermeulen et al., 2007). To test for specificity, we used as a control the inactive pseudo inhibitor compound also provided by the manufacturer. Effects on miR-2954 levels were tested 48 h after application of the inhibitor and control, using RT-qPCR (see below), and optimal inhibitor concentration was determined empirically using a dose-response analysis.

### Reverse transcription real-time quantitative PCR (RT-qPCR)

RNA samples were extracted from zebra finch tissues or cell lines by TRI Reagent (Ambion), treated with DNase (Ambion). The MicroRNA Assay Kit (Applied Biosystems) was used for reverse transcription and real-time qPCR of miR-2954 according to manufacturer's instructions as in a previous study (Gunaratne et al., 2011). The principle of the microRNA RT-qPCR was described by Chen et al. (2005). The primers for SYBR Green qPCR, designed using the Primer3 software (Rozen and Skaletsky, 1999), are given in Table 1. The RETROscript Kit (Ambion) and SYBR Green (Roche) qPCR was used to measure expression of nine predicted target mRNAs (Gunaratne et al., 2011; Table 2). As endogenous references we used the small RNA RNU6B for miR RT-qPCR and beta-actin for NR4A3 mRNA RT-qPCR.

**Table 1:** **Primers used for qRT-PCR analysis of predicted target genes**.

**Ensembl transcript**	**Gene**	**Forward primer**	**Reverse primer**
ENSTGUT00000009228	NR4A3	GTGGAATGTGAGTGGGAGGAA	TGGAGGACACAGACTACGTGAAA
ENSTGUT00000008541	BTG1	TGGGCTTCATCTCCAAGTTC	CCATCCTCTCCAATGCGATA
ENSTGUT00000008940	CHD2	TACAGACCAAACAACCTGTCC	GGAAATCCTGCTGGTGGTAG
ENSTGUT00000010614	CRKL	CTTCCCTCAGTATCCAGCAC	GACCGTTGACTTCTCCTTCC
ENSTGUT00000001410	ELAVL2	CATGGAAACACAACTGTCTAATG	AAACCGTAACCCAAACTCG
ENSTGUT00000012195	HMGB1	AAAGAAACTGGGAGAGATGTG	TCTAAAAGAGACTTATTCATCATCA
ENSTGUT00000010816	NEGR1	TCCCTTCTTACTGTTACCAATG	AGCCTTTTATGGGTCTTTACA
ENSTGUT00000001459	LINGO2	TCCAACGACACAAGTTCTAATG	TATTCAAGGTCAATGCTGGTT
ENSTGUT00000003233	TLK2	GATATTTTGCAGGAGAACACAA	ATACAGATTTGCGGATGTGAG

**Table 2:** **Differential expression testing of predicted target genes using RNA-seq and DE-Seq2**.

**Transcript ID**	**Gene**	**Mean reads**	**Log2FC**	***p*-val**	**Adj-*p***
ENSTGUT00000009228	NR4A3	53.9	−0.85	0.000	0.001
ENSTGUT00000008541	BTG1	1459.7	−0.16	0.012	0.038
ENSTGUT00000008940	CHD2	2296.7	−0.12	0.028	0.078
ENSTGUT00000010614	CRKL	151.8	−0.65	0.001	0.004
ENSTGUT00000001410	ELAVL2	9.6	−0.07	0.827	0.905
ENSTGUT00000012195	HMGB1	7222.7	−0.22	0.000	0.000
ENSTGUT00000010816	NEGR1	620.4	0.01	0.791	0.881
ENSTGUT00000001459	LINGO2	0	NA	NA	NA
ENSTGUT00000003233	TLK2	893.6	−0.12	0.133	0.277

### Fluorescence *in situ* hybridization (FISH) and immunohistochemistry (IHC)

To map miR-2954 expression within zebra finch brain at cellular and subcellular levels, we used fluorescence *in situ* hybridization (FISH) with a specific locked nucleic acid (LNA) probe. The sections were then double-stained with an antibody to the neuronal marker, NeuN, to distinguish neurons from non-neurons. A scrambled LNA sequence was used as negative control and generated no fluorescent signal.

Unlabeled LNA oligonucleotides (purchased from Exiqon) were labeled with digoxigenin (DIG) by the DIG Oligonucleotide Tailing Kit (Roche) and labeling efficiency was estimated using a dot blot. For a negative control, an LNA-modified, DIG-labeled scramble sequence was used. The protocol for using LNA probes and tyramide signal amplification (TSA) to detect microRNA in frozen tissue sections (Silahtaroglu et al., 2007) was followed and modified to include staining by a protein marker. Parasagittal sections, 10 μm in thickness, were collected beginning from midline to ~4.1 mm and alternatively placed on either a glass slide and stored at −80°C. Sections were removed from −80°C storage, fixed with 4% (wt/vol) paraformaldehyde (pH 7.6) for 5 min, treated with 0.25% (vol/vol) acetic anhydride/0.1 M triethanolamine for 10 min, and permeablized with 0.2% Triton X-100 for 15 min. *In situ* hybridization with 2.5 pmol DIG-labeled LNA probe in hybridization buffer [50% (vol/vol) formamide, 5× SSC, 500 μg/ml yeast tRNA, 1× Denhard's solution and DEPC treated water] was carried out for 3 h at 52°C followed by serial washes with saline-sodium citrate (SSC) buffers at 62°C. Sections were treated with 3% hydrogen peroxide to block endogenous peroxidase, washed with TN buffer (0.1 M Tris-HCl, pH 7.5 and 0.15 M NaCl) three times and then incubated in blocking buffer [0.1 M Tris-HCl, pH 7.5, 0.15 M NaCl, 0.5% (wt/vol) blocking reagent and 0.5% (wt/vol) BSA] for 30 min at room temperature or overnight at 4°C.

Following blocking, IHC was used to detect microRNA signals and neuronal markers. Sections were incubated by peroxidase-conjugated anti-DIG antibody (1:400 in blocking buffer; Roche) for 45 min and then washed three times with TNT buffer (0.1 M Tris-HCl, pH 7.5, 0.15 M NaCl, and 0.3% Triton-X-100). After washes, the DIG signals were amplified by the Cy5-tyramide Plus Kit (1:100; PerkinElmer) for 10 min. To label the neurons, sections were incubated with the primary antibodies against the neuronal marker, NeuN (1:500, MAB377; Millipore) for 1 h. After 1 h, tissues were washed with TNT buffer (5 min, 6 times) and incubated 1 h with the secondary antibodies Alexa 488 (1:500, A-21202; Invitrogen) and followed by TNT washes (6 times for 5 min). Slides were then dried and cover slip mounted with ProLong Gold Antifade Reagent with DAPI (P-36931; Invitrogen) for staining of cell nuclei. All IHC incubations were done at room temperature in a humidity chamber. Slides were imaged under a Zeiss LSM700 confocal microscope (Carl Zeiss Microimaging, Inc.).

### RNA sequencing

To test more broadly for genome-wide effects of manipulating miR-2954 in zebra finch cells, we applied RNA-seq to compare the population of mRNAs in the G266 cell line transfected with either the miR-2954 inhibitor or the control pseudo-inhibitor, which is predicted not to interact with any known zebra finch transcript. Total RNA samples were purified 48 h after transfection of miR-2954 inhibitor at 100 nM. The purified RNA samples were analyzed on Bioanalyzer (Agilent) to ensure adequate quality and quantity of RNA [RNA Integrity Number (RIN) > 8]. In total, six RNA-seq libraries were constructed, three per treatment, with Illumina's TruSeq RNA-seq Sample Prep kit following manufacturer's instructions. The libraries were quantitated by qPCR, pooled and sequenced on one lane for 100 cycles on an Illumina HiSeq2000 using a TruSeq SBS sequencing kit version 3 and analyzed with Casava pipeline 1.8. Library construction and sequencing were done at the University of Illinois Roy J. Carver Biotechnology Center.

Reads were mapped to the zebra finch reference genome (version 3.2.4) using Tophat version 2.0.9 (Trapnell et al., 2012). We used default settings, allowing reads to map up to 20 times, and we based differential expression tests on this subset of reads. The resulting BAM file from the Tophat mapping was converted to SAM format using SAMtools, and a table of mapped read counts per gene was derived using Ensembl gene annotations (version 73) and HT-seq (version 0.4.5p5) (Anders et al., 2014). We tested for differential expression using DE-seq2 (version 1.0.19) (Anders and Huber, 2010). DE-Seq2 models variance in gene expression as count data using a negative binomial distribution, which has been shown to correspond well with the observed relationship between expression level and variance (Anders and Huber, 2010). The modeled variance expectation is based both on the observed variability in the gene being tested and the distribution of variance of genes in the rest of the genome. DE-Seq is similar to other popular statistical tests for differential expression (e.g., edge-R; Robinson et al., 2010) and has been demonstrated to be reliable, with a reasonable balance of power and false discovery rate (e.g., Ching et al., 2014; Robinson and Storey, 2014; Zhou et al., 2014). The test compared the mapping profile of three replicate control libraries and three libraries that received the inhibitor treatment. To examine the functional representation of the differentially expressed transcripts, we used Gene Ontology (GO) and Kyoto Encyclopedia of Genes and Genomes (KEGG) analyses (http://www.ark-genomics.org/tools/GOfinch, http://www.ark-genomics.org/tools/KEGGfinch; Wu and Watson, 2009). Fisher's exact tests were used to test for statistical overrepresentation of GO categories and KEGG pathways among lists of differentially expressed genes.

## Results

### Variation in miR-2954 expression by sex and tissue

**Figure 1:**
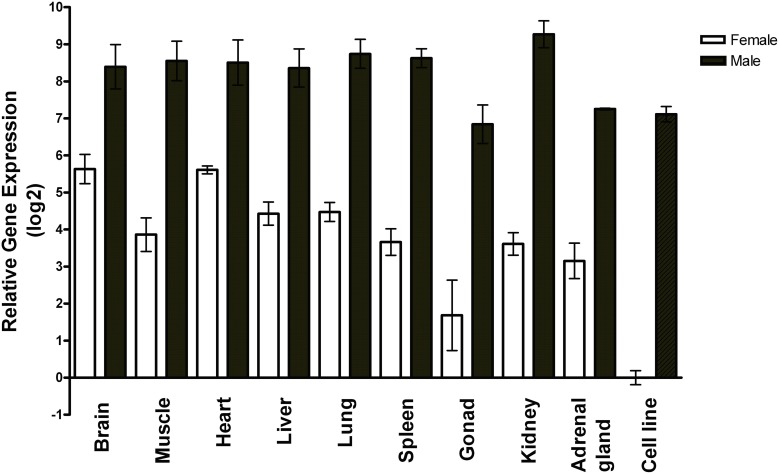
**Expression profile of miR-2954 in nine major tissues and two cell lines of zebra finch**. Each bar represents the mean and standard error of expression measured by RT-qPCR, shown as the log2 of the fold-difference relative to the female cell line. Thus, the value for the female cell line itself (Ct mean: 27 cycles) is log2(1) = 0. Each tissue value represents the mean of samples from three separate birds for each sex. Each cell line value represents the mean of three separate culture dishes.

Using RT-qPCR, we measured miR-2954 in two non-neural zebra finch cell lines and nine zebra finch tissues, and found it to vary by only ±2-fold among the tissues (Figure 1). A sex-biased expression pattern is evident in all tissues, with lower expression in females by 10-fold or more, and roughly 100-fold lower expression in the female ZFTMA cell line (derived from a tumor found on the thigh) compared to the male G266 line [derived from a tumor found on the forehead (Itoh and Arnold, 2011)]. The ubiquitous expression pattern across different tissues and higher expression levels in males is consistent with observations made using various techniques in both chicken (Zhao et al., 2010) and zebra finch (Luo et al., 2012).

### Cellular and subcellular localization in male song nuclei

Using *in situ* hybridization of zebra finch brain sections, we detected expression broadly throughout the brain, and focused our analysis on the major regions of the telencephalon involved in song production and perception: HVC (letters used as proper name), the striatal nucleus Area X, the lateral magnocellular nucleus of the anterior nidopallium (LMAN), the robust nucleus of the arcopallium (RA), and the caudomedial nidopallium (NCM). HVC, Area X, LMAN, and RA are responsible for song production in males and are absent or much reduced in females whereas NCM (in the auditory forebrain) is morphologically similar in both sexes.

**Figure 2:**
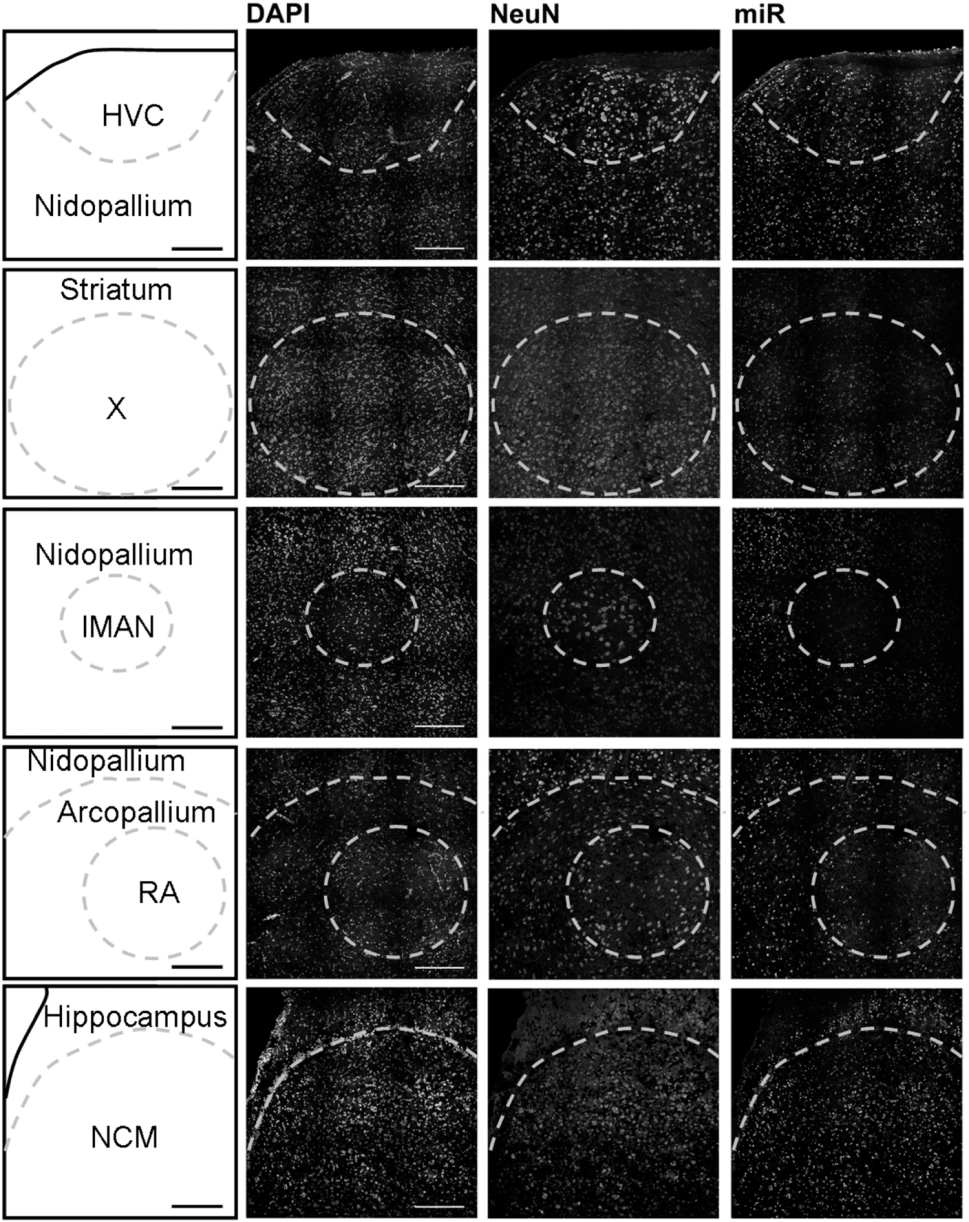
**Mapping of miR-2954 expression in zebra finch song nuclei (overview)**. Low-magnification view of miR-2954 expression in HVC, Area X, the lateral magnocellular nucleus of the anterior nidopallium (LMAN), the robust nucleus of the arcopallium (RA), and the caudal medial nidopallium (NCM). Scale bar represents 0.2 mm.

**Figure 3:**
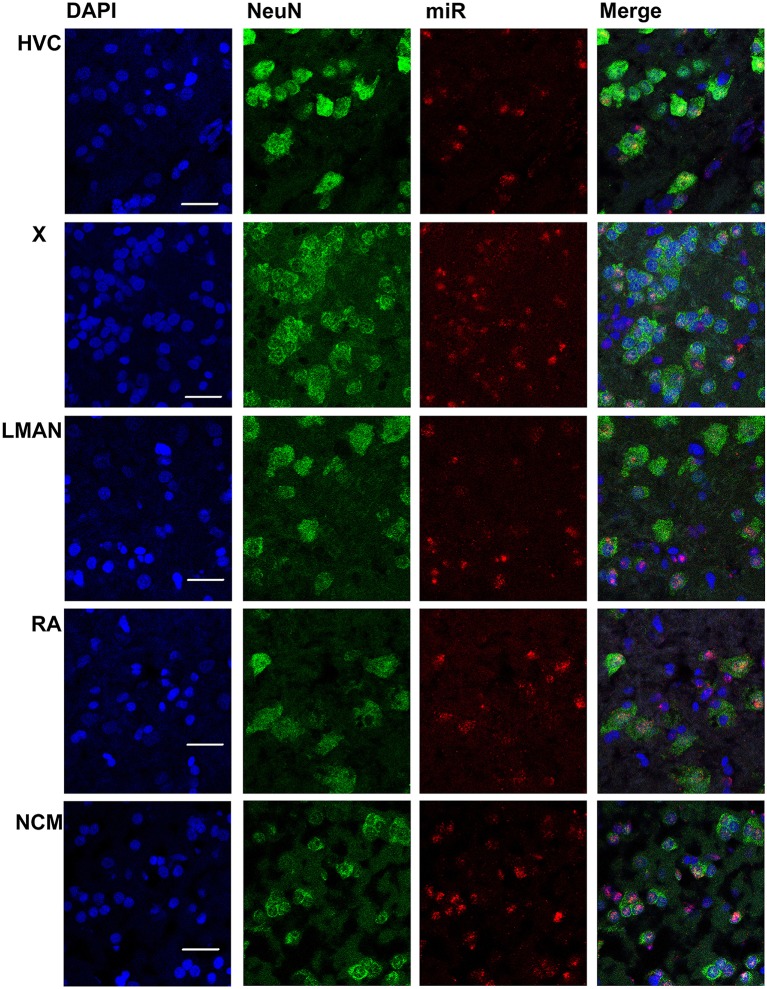
**Mapping of miR-2954 expression in zebra finch song nuclei (zoom in)**. High-magnification images in the song nuclei show the overlapping of miR-2954, DAPI (labeling all nuclei), and NeuN (labeling neurons). Scale bar represents 0.02 mm.

In males, HVC is readily distinguished from the surrounding nidopallium by its concentration of large neurons, evident at low magnification by Nissl staining (not shown) or by immunocytochemistry for NeuN (Figure 2). The density of miR-2954-positive cells is similar in both the HVC interior and the surrounding nidopallium (Figure 2). At higher magnification, the miR-2954 signal is typically concentrated in small domains that appear to lie within or immediately adjacent to DAPI-positive nuclei (Figure 3, compare blue DAPI and red miR-2954) inside larger cell bodies that are co-labeled for NeuN (Figure 3, compare green NeuN, and also merged image). Only about half of the NeuN-positive cells are double-labeled for miR-2954, indicating that miR-2954 is present in only subsets of neurons. HVC has two classes of projection neurons and the larger neurons project to Area X (Nixdorf et al., 1989; Fortune and Margoliash, 1995; Dutar et al., 1998); based on the large soma size of miR-2954-positive/NeuN-positive figures, we suggest neuronal expression may be limited to the X-projecting subset although additional evidence (e.g., retrograde tracer analysis) would be needed to confirm this. A few miR-2954-positive cells can also be seen in cells that are not NeuN-positive, suggesting probable expression in a subset of glial cells, although this would also need to be confirmed with additional markers.

Area X itself is characterized by a dense population of relatively small neurons, and miR-2954 is present generally in this population (Figures 2, 3). In contrast, LMAN is characterized by a core region of large neuronal cell bodies, and this core region contains little or no miR-2954 (Figure 2). Figure 3 reveals an absence of miR-2954 in the large central core neurons of LMAN. Some labeling is evident in the surrounding shell region, possibly including some non-neuronal cells (one apparent example of non-neuronal labeling in the LMAN shell can be seen in Figure 3). Nucleus RA is also characterized by a central population of large neurons, and these also show partial overlap for miR-2954 (Figures 2, 3).

In the auditory region NCM, miR-2954 labeling is apparent throughout, though somewhat less robust in the ventral region immediately beneath the overlying ventricle (Figure 2). Cells in NCM are often arranged in grape-like clusters in which neurons and glia are hard to resolve; some of these clusters show little or no labeling, and in other cases only one or two cells of a cluster appear to express mir-2954 (Figure 3). We also considered whether miR-2954 localization in NCM might change as a result of song playbacks or vary with tissue sex. We did not observe any evidence for this by FISH, however (Supplementary Material: Figures S1, S2).

In sum, miR-2954 labeling is most apparent within cell nuclei where it is often further concentrated in puncta or subregions suggestive of nucleoli. It is found in distinct neuronal subpopulations in the song system (e.g., present in HVC and Area X, less so in RA and absent from the large neurons of LMAN core), and occasionally detected in cells that appear to be non-neuronal by absence of NeuN immunostaining.

### Validation of miR-2954 knock-down in cultured cells

**Figure 4:**
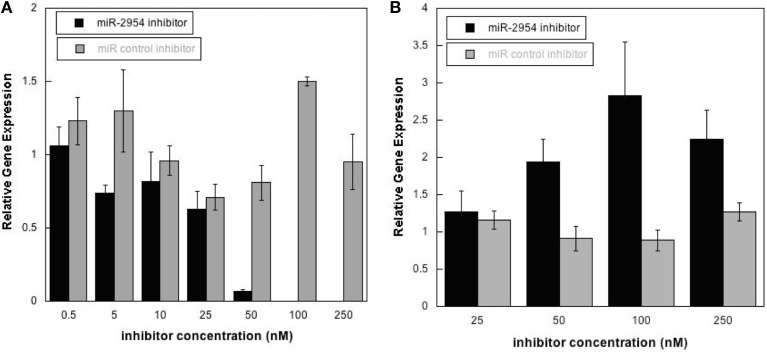
**(A)** Effects of miR-2954 inhibitor or control dosage on miR-2954 levels in G266 cells, measured by RT-qPCR (linear scale). **(B)** Effects of miR-2954 inhibitor or control dosage on NR4A3 mRNA levels in G266 cells, measured by RT-qPCR (linear scale).

To test the hypothesis that miR-2954 affects the concentration of target mRNAs, we depleted miR-2954 levels in the G266 cell line, where miR-2954 is normally abundant (Figure 1). Transfection of a miR-2954 inhibitor at increasing concentrations resulted in a significant 10-fold decrease in miR-2954 (ANOVA, *p* < 1E-7) when the inhibitor concentration reached 50 nM, and miR-2954 was undetectable in cells treated with inhibitor at 100 nM. The control compound had no significant effect (ANOVA *p* > 0.99) on miR-2954 at concentrations up to 250 nM (Figure 4).

Inhibition of miR-2954 also resulted in a significant increase (ANOVA *p* = 4.5E-5), in the mRNA for NR4A3 (Nuclear receptor subfamily 4 group A member 3), a major predicted target of miR-2954 (Warren et al., 2010; Gunaratne et al., 2011) (Figure 4). Again, the control pseudo inhibitor had no significant effect on NR4A3 mRNA. *Post-hoc* tests (*p* = 0.020 at 50 nM inhibitor; *p* = 0.001 at 100 nM inhibitor *p* = 0.007 at 250 nM inhibitor) established 100 nM as an optimal inhibitor concentration that reduces miR-2954 levels, leading to the increase in target mRNAs that are normally suppressed by the microRNA. We did not detect an effect of the miR-2954 inhibitor on eight other predicted target genes via qRT-PCR (BTG1, CHD2, CRKL, ELAVL2, HMGB1, NEGR1, LINGO2, and TLK2; but see RNA-seq results below). One of these, LINGO2, could not be detected in either of the two cell lines.

### Consequences of miR-2954 knock-down on genome-wide expression profiles

To test more broadly for consequences of miR-2954 knock-down on gene expression, we collected RNAs from G266 cultures that had been treated with either the inhibitor compound or the control (100 nM; each condition in triplicate), and performed Illumina RNA-seq analysis. The six libraries contained an average of 24,156,792 reads per library. Seventy percent of these reads mapped to the genome under our mapping settings in TopHat. RNAs from 15,289 of 18,618 Ensembl (v73)-annotated genes were detected with at least one read in at least one of the libraries (Supplementary Material: Table S1). Using DE-Seq2, we detected 3509 genes that were differentially expressed between the two treatments at an adjusted *p*-value less than 0.01 (Benjamini and Hochberg, 1995). The predicted target, NR4A3 was among these genes (adjusted *p* < 0.001; Table 2). Three of the seven other predicted target genes were also significant after correction for multiple testing at *p* < 0.05 (BTG1, CRKL, HMGB1) and like NR4A3, all were expressed at a higher level following miR inhibition. A fifth predicted target gene (CHD2) bordered on statistical significance and was also more highly expressed following inhibitor treatment (Table 2).

**Figure 5:**
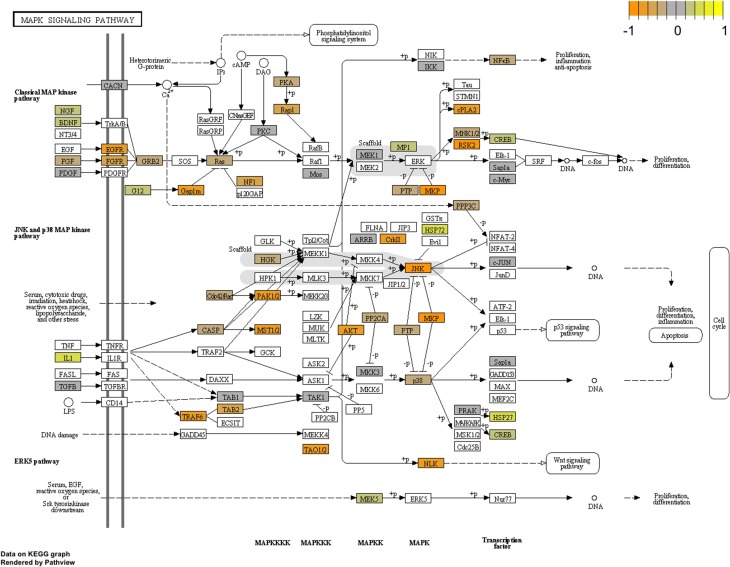
**Mapping of RNA-seq data to MAPK pathway (KEGG)**. The figure shows the complete pathway as annotated in KEGG, but shading (orange, gray, yellow) is applied only to those RNAs detected in our dataset that also pass DESEQ2 statistical filters and map to a unique ENTREZ ID. Color corresponds to magnitude and direction of log2 fold change (control/inhibitor), with orange (negative fold change) indicating genes that are suppressed by miR-2954 (i.e., increase in expression after experimental inhibition of mir-2954).

To arrive at a more stringently qualified list of gene responses to inhibition of mir-2954, we applied an additional criterion for magnitude of effect, filtering the list above for only those genes that were up-regulated by at least 1.5 fold (864 genes), or suppressed by at least 0.75-fold (232 genes) after application of the inhibitor (Supplementary Material: Table S2). We then performed GO and Kyoto Encyclopedia of Genes and Genomes (KEGG) analyses by comparing these lists of significantly regulated genes against the entire population of 14,987 whose expression was detected and passed filtering in DE-Seq2 (Tables 3, 4). Among the up-regulated genes (i.e., those that increase following miR-2954 inhibition and therefore must be directly or indirectly suppressed by mir-2954 expression), genes associated with serine/threonine and tyrosine protein kinase signaling function are strongly over-represented (with adjusted *p-values* < 0.0001, Table 3A), whereas genes in the G-protein coupled receptor protein signaling pathway (GO:0007186) are significantly underrepresented (adjusted *p* = 0.023, Table 3A). KEGG pathway analysis (Table 4) specifically implicates the MAPK pathway in the overrepresented gene set: 22 (2.5%) are annotated for the MAPK pathway, even though MAPK genes comprise only 118 (0.6%) of all genes in the genome. The MAPK pathway is illustrated in Figure 5, with color-coding to indicate magnitude and direction of expression changes after miR-2954 inhibition, for all MAPK-related genes detected in our data. Normalized RNA-seq read counts for all six individual samples underscore the magnitude and reproducibility of the effect of miR-2954 inhibition on MAPK-related genes (Figure S3).

**Table 3:** **Gene Ontology (GO) analysis of the response to mir-2954 inhibition**.

**GO ID**	**Description**	**Expected/Observed**	**Adjusted *P***
**(A)**			
0005515	Protein binding	315/394	1.50E-06
0004713	Protein tyrosine kinase activity	23/52	1.10E-05
0004674	Protein serine/threonine kinase activity	18/45	1.20E-05
0006468	Protein phosphorylation	30/58	0.00032
0018107	Peptidyl-threonine phosphorylation	1/8	0.00058
0016772	Transferase activity, transferring phosphorus-containing groups	28/55	0.00058
0004672	Protein kinase activity	27/52	0.00150
0005543	Phospholipid binding	14/32	0.00160
0035329	Hippo signaling cascade	0/5	0.00340
0043547	Positive regulation of GTPase activity	3/12	0.00410
0005096	GTPase activator activity	3/11	0.01000
0007186	G-protein coupled receptor signaling pathway^*^	19/5	0.02300
0016567	Protein ubiquitination	7/17	0.02900
0004842	Ubiquitin-protein ligase activity	8/19	0.03600
0055038	Recycling endosome membrane	1/5	0.03700
0012506	Vesicle membrane	0/4	0.04900
0046777	Protein autophosphorylation	4/12	0.04900
0008270	Zinc ion binding	70/98	0.0500
**(B)**			
0003735	Structural constituent of ribosome	2/21	2.1E-15
0005840	Ribosome	2/21	2.1E-15
0006412	Translation	2/21	1.1E-13
0005739	Mitochondrion	10/38	1.2E-10
0030529	Ribonucleoprotein complex	1/11	1.5E-05
0008121	Ubiquinol-cytochrome-c reductase activity	0/4	0.00016
0005515	Protein binding^*^	68/40	0.00029
0022625	Cytosolic large ribosomal subunit	0/5	0.0041
0005758	Mitochondrial intermembrane space	0/3	0.0041
0005680	Anaphase-promoting complex	0/3	0.028

*Frequency of annotation terms was analyzed for (A) 864 up-regulated genes (i.e., miR-suppressed, >1.5-fold) and (B) 232 down-regulated genes (i.e., miR-supported, <0.75-fold). The observed frequency of each GO term is contrasted with the expected (chance) frequency, and the probabilities in the table are adjusted for multiple testing (Benjamini–Hochberg adjusted p < 0.05)*.

* *Denotes significantly under-represented GO terms (all other terms are over-represented)*.

**Table 4:** **Enrichment of KEGG pathways in up- and down-regulated gene sets (following miR-2954 inhibition)**.

**KEGG ID**	**Pathway name**	**Expected/Observed**	**Adjusted-*P***
**Up-regulated (i.e., miR-suppressed)**			
gga04010	MAPK signaling pathway	7/22	0.00049
**Down-regulated (i.e., miR-supported)**			
gga03010	Ribosome	1/9	2.20E-06
gga00190	Oxidative phosphorylation	1/7	0.00190

A very different functional profile is seen for the set of genes that decrease in expression after miR-2954 inhibition, with strong enrichment of terms for ribosome, translation and mitochondria as seen both by GO analysis (Table 3B) and KEGG analysis (Table 4). The effect on ribosomal genes is even more striking when the entire dataset is considered without filtering for magnitude of change: 50 of 51 ribosomal genes (all significant at adj *p* < 0.01) are now included. That is to say, essentially all genes that encode ribosomal proteins are sensitive to a reduction in miR-2954, decreasing their expression as a consequence.

### Comparison with the response to song playback

The decrease in expression of genes annotated for ribosome and mitochondrial functions (Tables 3, 4) is reminiscent of a functional change in gene expression in the auditory forebrain that follows a day after repeated playbacks (Dong et al., 2009), which also causes a decrease in miR-2954 expression in the male brain (Gunaratne et al., 2011). We therefore looked specifically for overlap between the gene sets sensitive to miR-2954 inhibition or to song stimulation, using the data from the previous song playback studies. Fifty one genes show significant effects of both treatments, with response magnitudes of at least 1.5-fold up or down (Table S3), with 36 decreasing after both song stimulation and miR-2954 inhibition. At least a third of these have clear functions in translation and mitochondrial energetics (Table S3).

## Discussion

Here we described experiments to assess the potential functional significance in the zebra finch brain of miR-2954, a novel sex-linked microRNA found so far only in birds. Our approach involved consideration of both the anatomical distribution of miR-2954 in the zebra finch brain (using high-resolution FISH) and the consequences of miR-2954 inhibition in cultured zebra finch cells using RNA-seq to profile the complex genomic response. The zebra finch is emerging as a model for analysis of gene networks involved in cognition (Clayton, 2013) but this is the first study to profile the consequences of microRNA manipulation on endogenous gene expression in zebra finch cells.

Although miR-2954 is broadly expressed across many tissues, in the brain its expression is not ubiquitous and it shows discrete localization to subsets of brain cells. We paid special attention to its expression in the major well-defined, sexually dimorphic nuclei of the song control system. There we observed predominant but not exclusive expression in neurons. The miR is found in different neuronal subsets in the different song nuclei, being conspicuously absent from the large magnocellular neurons of lMAN yet present in large neurons in HVC. In general the miR is expressed at similar levels within the song nuclei compared to surrounding brain regions. Hence miR-2954 could clearly play a role in vocal behaviors but is likely to have other roles as well.

At the subcellular level, miR-2954 appears to be localized primarily to the cell nucleus. Although this contrasts with the common expectation that microRNAs function primarily in the cytoplasm, most microRNAs are also present in the nucleus and evidence is accumulating that some microRNAs have discrete functions in the nucleus (reviewed in Liang et al., 2013; Roberts, 2014). These nuclear functions are not yet well understood but may involve epigenetic regulation of gene expression through interactions with long ncRNAs. A systematic attempt to characterize the population of nuclear microRNAs in cultured rat neurons identified two in particular that are enriched in the cell nucleus, miR-25 and miR-92 (Khudayberdiev et al., 2013). Intriguingly, both of these were also identified as song-responsive in our previous RNA-seq analysis, with miR-25 increasing and miR-92 decreasing upon song exposure (Gunaratne et al., 2011). We speculate that all these song-responsive miRs may all participate in some nuclear epigenetic process in the auditory forebrain that is triggered by the acute experience of hearing song playback after social isolation.

The mir-2954 gene maps to the Z chromosome and thus is present in twice as many copies in males (ZZ) than in females (ZW). In birds, due to incomplete dosage compensation, Z-linked genes typically produce more RNA in males than in females (Ellegren et al., 2007; Itoh et al., 2007). Higher expression of miR-2954 in males has previously been shown for brain tissue (Gunaratne et al., 2011) and in chicken for various other tissues (Zhao et al., 2010), and here we also measured higher male expression in various tissues of the zebra finch. We found the sex difference in miR-2954 to be much more than 2-fold in most tissues, however, indicating that factors other than gene dosage must amplify expression specifically in males or suppress it specifically in females. For example, expression of the gene could be sensitive to circulating gonadal steroids or affected by various rates of transcription, processing of pri-miRNA and pre-miRNA, or stability of mature miRNA in different tissues. However, we detected an expression difference of 100-fold in two zebra finch cell lines that differ in chromosomal sex. In this case, the expression difference must be due to cell-autonomous factors and not to the influence of extrinsic regulatory signals. This suggests that miR-2954 participates in a transcriptional regulatory network that amplifies intrinsic sex differences in gene dosage for some genes.

The mir-2954 gene lies within an intron of the XPA gene. We do not yet know whether there is functional or regulatory significance to this relationship, but mutations in XPA have been associated with neurodegenerative conditions (Taylor, 2008; Tomasevic et al., 2012). Many miRs are processed from introns of other genes, potentially affording multiple levels of control over production of the miR. Here we focused only on the primary mir-2954 sequence, but note that other sense and antisense sequences also arise from the mir-2954 gene locus (Figure 2 in Gunaratne et al., 2011) and could have functional significance though we have not yet explored that possibility.

To gain insight into the biological function of miR-2954, we used a new zebra finch cell line which, although derived from non-neural tissue, does express a large number of neural genes (Balakrishnan et al., 2012). Observations made in this cell line may not necessarily generalize to the intact brain, but cell line studies represent a tractable first step toward more ambitious future research to manipulate miRs in the zebra finch brain itself. A lack of such cell lines has limited molecular biology research on zebra finches to date. Using a commercially supplied sequence-specific miR-2954 inhibitor, we were successful in reducing the endogenous expression of miR-2954 in the zebra finch G266 cell line (Figure 4A). This resulted in a significant increase in expression of one of the predicted targets of miR-2954, the transcription factor gene NR4A3 (Figure 4B). NR4A3 is notable as one of the most strongly responsive mRNAs in song stimulation experiments (Dong et al., 2009; Warren et al., 2010). However, using quantitative RT-PCR, we did not detect an effect on eight other potential target mRNAs predicted by Targetscan.

Therefore, we went forward with an unbiased genome-wide analysis to assess consequences of miR-2954 inhibition, using RNA-seq technology (Mortazavi et al., 2008; Singh et al., 2011; Tarazona et al., 2011; Zhou et al., 2011). RNA-seq was recently used in the primary characterization of both of the two new zebra finch cell lines (Balakrishnan et al., 2012). Here we observed statistically significant changes in the expression of 3509 mRNAs after application of the miR-2954 inhibitor to the G266 (male) cell line. These again included an increase in NR4A3, plus three of the targets previously predicted by Targetscan (with two other predicted targets showing trends that fell short of significance). However, most of these measured changes were of small magnitude and so we focused our further analysis on the effects of larger magnitude (increases by at least 1.5-fold, or decreases by at least 0.75-fold), which still resulted in a list of 1096 genes. It is important to note that these genes are not necessarily targets of direct molecular regulation by miR-2954; all we can say is that they represent a profile of how cells respond when miR-2954 is inhibited. By studying this response we hope to gain insight into the larger biological function of miR-2954 as just one element in a complex dynamic system.

Given such a large number of responding genes, we applied a population approach to infer possible functional consequences by looking for enrichment of GO and KEGG pathway annotation terms in the responding set. We took a similar approach in a previous microarray study which identified a similarly large number of genes that respond to song playback (Dong et al., 2009). As in this previous study, we found striking differences in the functional profiles of the genes that increased in expression, compared to those that decreased. Among the increasing mRNAs, the MAPK pathway emerged as the dominant signature: nearly 20% of all MAPK-pathway-related genes increased in expression by at least 1.5-fold after the inhibitor was applied. This effect was quite specific to the MAPK pathway, as no other KEGG pathway reached statistical significance and genes with GO annotations for the G-protein signaling pathway are specifically under-represented (Table 3). Among the mRNAs that decreased, a completely different functional profile was evident, with statistically robust enrichment for genes involved in ribosome structure, translation, and mitochondrial energetics. We infer that, under the basal conditions of this cell line, endogenous miR-2954 expression tends to suppress MAPK gene expression and to support the expression of genes involved in energetics and protein biosynthesis. We note again that these effects of miR-2954 on the gene expression networks do not have to be direct. Rather, a change in miR-2954 leads (whether directly or indirectly or both) to a complex but specific set of changes in the structure of the cell's transcriptional network.

Song exposure also causes changes in miR-2954 expression in the auditory forebrain and therefore comparison with the cell line data presented here is of interest. In the AL, novel song exposure initially causes a decrease in miR-2954 (Gunaratne et al., 2011). A day after song exposures, the functional profile of gene expression has changed profoundly, with marked decline in mRNAs annotated for ribosomes and mitochondrial function (the “Habituate Down” profile, Dong et al., 2009). This is strikingly similar to the suppression of ribosomal and mitochondrial genes observed here in the G266 cell line after 2 days of exposure to the miR-2954 inhibitor. We confirmed that many of these genes are the same in the two datasets (Table S3). Thus, it is plausible to speculate that the song-stimulated decrease in miR-2954 may contribute to the process of song habituation in the brain. In future research it would interesting to evaluate whether these overlapping response genes also show a sex difference in the response to birdsong, paralleling the evident sex difference in the miR-2954 response itself. If so, this would strengthen the connection between miR-2954 and sex differences in neurogenomic networks.

Natural experience elicits complex changes in gene expression in parts of the brain associated with perception and cognition. Though early studies focused on just a few genes that are broadly responsive to cellular signals (the immediate early genes or IEGs), high-throughput techniques have now revealed that hundreds of genes may vary in their expression depending on the experience, environment, brain system and species (Cavallaro et al., 2002; Whitfield et al., 2003; O'Sullivan et al., 2007; Oldham et al., 2008; Dong et al., 2009; London et al., 2009; Mukai et al., 2009; Ellis and Carney, 2011; Drnevich et al., 2012; Ramsey et al., 2012). By what logic are these different gene networks organized, and how do they evolve? Regulation through microRNAs represents one mechanism that may coordinate the expression of many genes at once and allow the rapid evolution of new patterns of coordinate gene regulation.

Parallels between the microRNA manipulation in cells and the observations in zebra finches suggest some broad conclusions and important directions for future investigation. A single targeted miR manipulation can bring about large changes in ribosomal and mitochondrial gene expression—and in a pattern that shows similarities to the effects of perceptual experience in the brain. This suggests that there may be an integrated molecular system or pathway that links an organism's perceptual experience to broad regulation of energy metabolism—something that could clearly have long-term adaptive value to the organism. MiR-2954 may represent an important node in such a network, at least in birds. Future studies aimed at testing specifically for changes in energetics (e.g., using fMRI or specific metabolic indicators) following either miR manipulation or song exposure would be informative. Our results also suggest that the overwhelming complexity of gene regulation after natural experience might be reducible to a smaller subset of functional modules, which might be isolated and studied even in non-neural cell lines. Finally, our results here support the drive toward systems biology approaches even in non-traditional model organisms emphasizing evolving networks of interacting genes (Miyashita et al., 2008; Robinson et al., 2008; Clayton, 2013) as opposed to the deterministic linear cascade models that have dominated molecular neurobiology in the past.

## Conflict of interest statement

The authors declare that the research was conducted in the absence of any commercial or financial relationships that could be construed as a potential conflict of interest.

## Supplementary material

Figure S1 **Low magnification overview of miR-2954 expression in the auditory forebrain of zebra finch**.

Figure S2 **Cellular localization of miR-2954 expression in the auditory forebrain of zebra finch**.

Figure S3 **Normalized RNA-seq read counts from each of the six cell culture samples are plotted for nine of the statistically significant MAPK-related genes (Figure 5)**.

Table S1 **Differential expression analysis (all mapped RNA-seq reads)**.

Table S2 **Differential expression analysis (minimum 1.5-fold change)**.

Table S3 **Overlap analysis: genes that respond to both song stimulation (auditory forebrain) and miR-2954 inhibition (G266 cell line)**.
