# Supplementary material for: Functional genomic analysis and neuroanatomical localization of miR-2954, a song-responsive sex-linked microRNA in the zebra finch
Source: Front Neurosci. 2014 Dec 16;8:409. doi: 10.3389/fnins.2014.00409 (PMC4267206; doi:10.3389/fnins.2014.00409)
Supplement: Figure S1 — Low magnification overview of miR-2954 expression in the auditory forebrain of zebra finch. [file Image1.PDF]

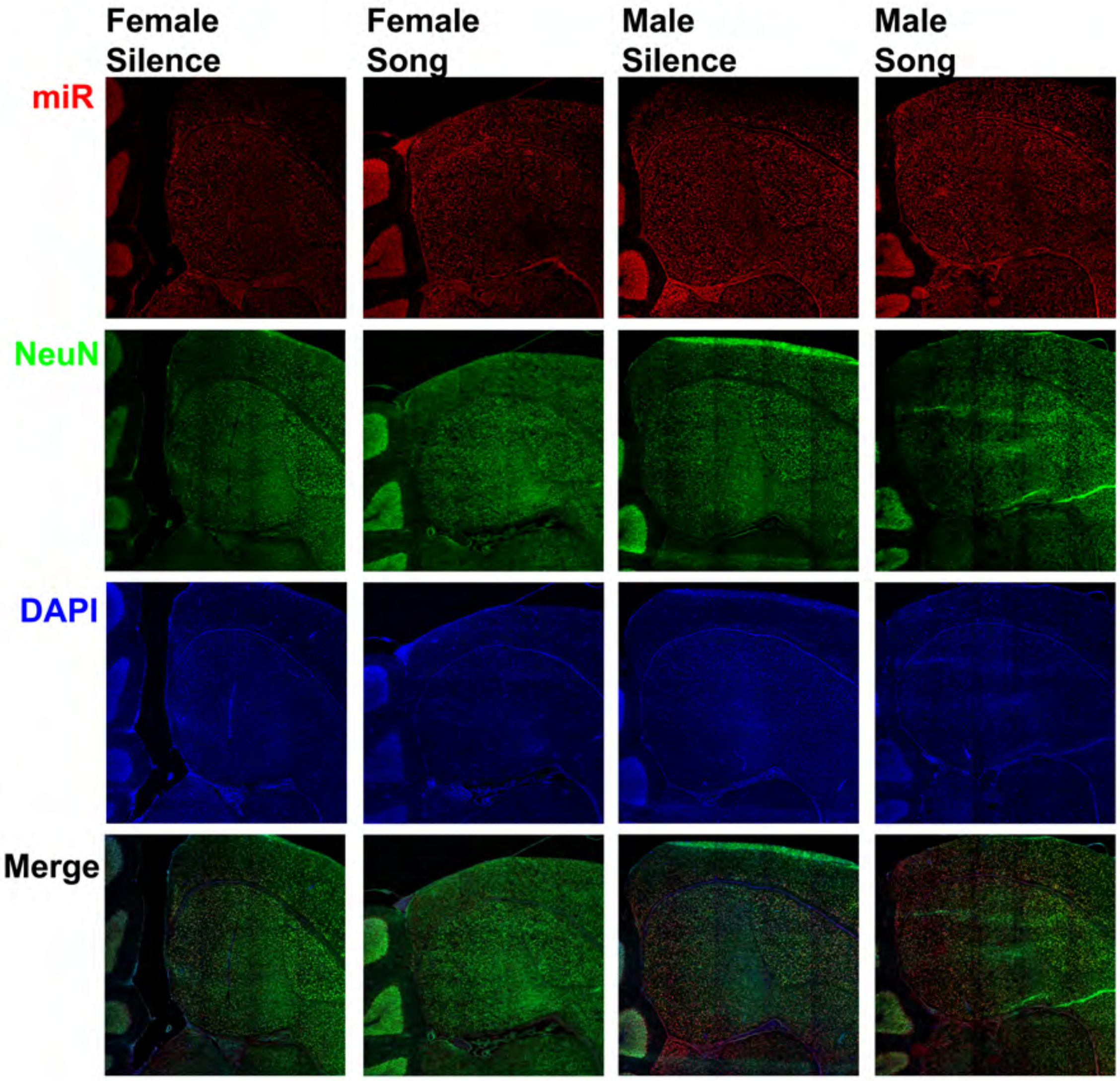


**Figure S1. Low magnification overview of miR-2954 expression in the auditory forebrain of zebra finch.** Representative images comparing labeling patterns in sagittal sections of individual birds (sex as indicated) either in a sound isolation chamber (Silence) or 30 min after onset of tape-recorded song playback (Song). Sections are labeled as in Figure 2.
