# Supplementary figures and images for: Functional genomic analysis and neuroanatomical localization of miR-2954, a song-responsive sex-linked microRNA in the zebra finch
Source: Front Neurosci. 2014 Dec 16;8:409. doi: 10.3389/fnins.2014.00409 (PMC4267206; doi:10.3389/fnins.2014.00409)

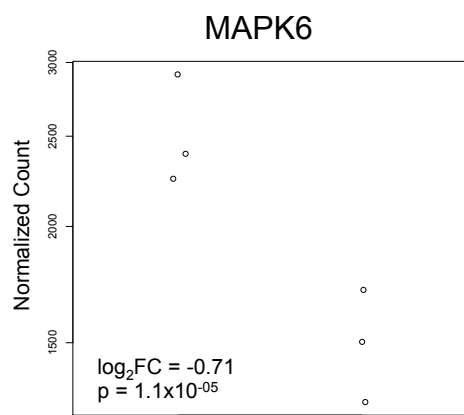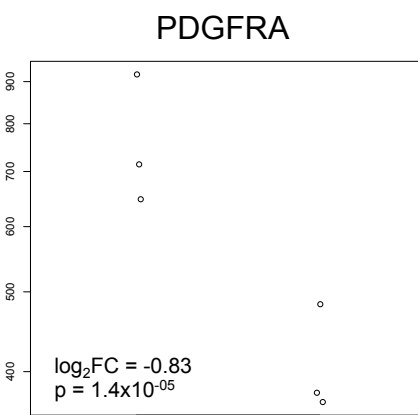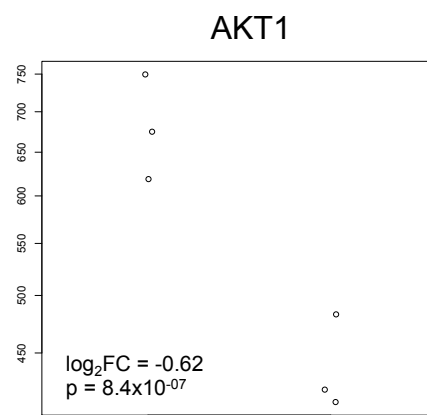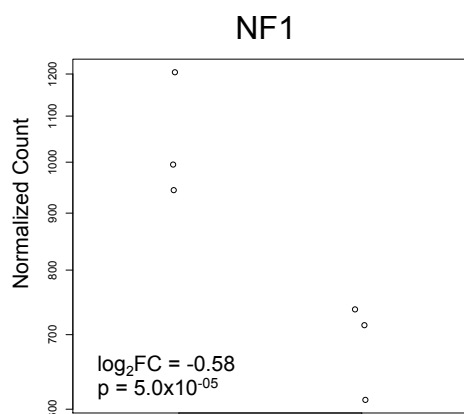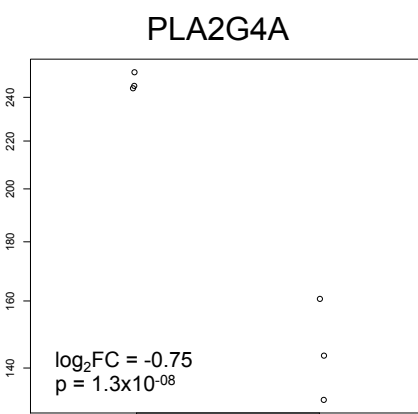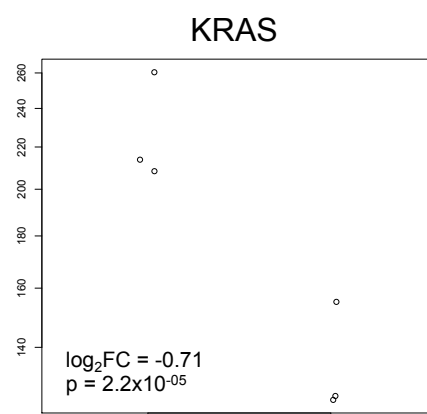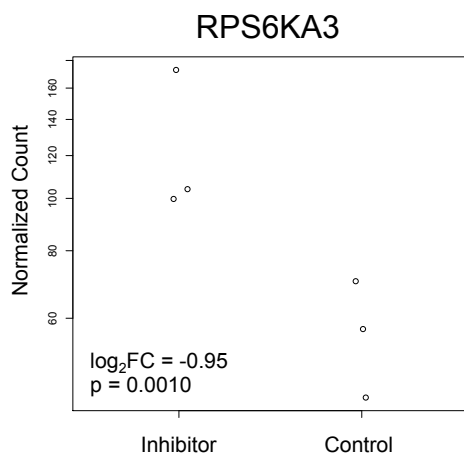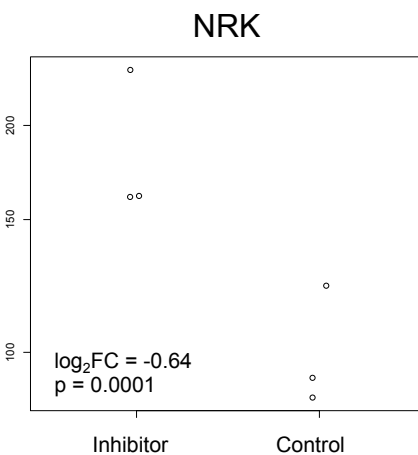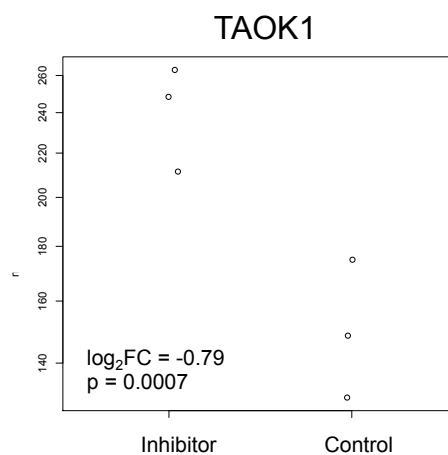

Supplement: Figure S3 — Normalized RNA-seq read counts from each of the six cell culture samples are plotted for nine of the statistically significant MAPK-related genes (Figure 5). [file Image3.PDF]
